# Mechanisms behind changes of neurodegeneration biomarkers in plasma induced by sleep deprivation

**DOI:** 10.1093/braincomms/fcad343

**Published:** 2023-12-12

**Authors:** Per Kristian Eide, Aslan Lashkarivand, Are Hugo Pripp, Lars Magnus Valnes, Markus Hovd, Geir Ringstad, Kaj Blennow, Henrik Zetterberg

**Affiliations:** Department of Neurosurgery, Oslo University Hospital—Rikshospitalet, N-0424 Oslo, Norway; Institute of Clinical Medicine, Faculty of Medicine, University of Oslo, N-0316 Oslo, Norway; Department of Neurosurgery, Oslo University Hospital—Rikshospitalet, N-0424 Oslo, Norway; Institute of Clinical Medicine, Faculty of Medicine, University of Oslo, N-0316 Oslo, Norway; Oslo Centre of Biostatistics and Epidemiology, Research Support Services, Oslo University Hospital, N-0424 Oslo, Norway; Faculty of Health Sciences, Oslo Metropolitan University, N-0130 Oslo, Norway; Department of Neurosurgery, Oslo University Hospital—Rikshospitalet, N-0424 Oslo, Norway; Section for Pharmacology and Pharmaceutical Biosciences, Department of Pharmacy, University of Oslo, N-0316 Oslo, Norway; Department of Transplantation Medicine, Oslo University Hospital, N-0424 Oslo, Norway; Department of Radiology, Oslo University Hospital—Rikshospitalet, N-0424 Oslo, Norway; Department of Geriatrics and Internal medicine, Sorlandet Hospital, N-4836 Arendal, Norway; Department of Psychiatry and Neurochemistry, Institute of Neuroscience and Physiology, the Sahlgrenska Academy at the University of Gothenburg, S-405 30 Gothenburg, Sweden; Clinical Neurochemistry Laboratory, Sahlgrenska University Hospital, S-405 30 Gothenburg, Sweden; Department of Psychiatry and Neurochemistry, Institute of Neuroscience and Physiology, the Sahlgrenska Academy at the University of Gothenburg, S-405 30 Gothenburg, Sweden; Clinical Neurochemistry Laboratory, Sahlgrenska University Hospital, S-405 30 Gothenburg, Sweden; Department of Neurodegenerative Disease, UCL Institute of Neurology, Queen Square, London WC1E 6BT, UK; UK Dementia Research Institute at UCL, London WC1E 6BT, UK; Hong Kong Center for Neurodegenerative Diseases, Clear Water Bay, Hong Kong 999077, China; Department of Medicine, UW School of Medicine and Public Health, Madison, WI 53726, USA

**Keywords:** acute sleep deprivation, brain metabolism, amyloid-β, molecular clearance, CSF-to-blood clearance

## Abstract

Acute sleep deprivation has been shown to affect cerebrospinal fluid and plasma concentrations of biomarkers associated with neurodegeneration, though the mechanistic underpinnings remain unknown. This study compared individuals who, for one night, were either subject to total sleep deprivation or free sleep, (i) examining plasma concentrations of neurodegeneration biomarkers the morning after sleep deprivation or free sleep and (ii) determining how overnight changes in biomarkers plasma concentrations correlate with indices of meningeal lymphatic and glymphatic clearance functions. Plasma concentrations of amyloid-β 40 and 42, phosphorylated tau peptide 181, glial fibrillary acid protein and neurofilament light were measured longitudinally in subjects who from Day 1 to Day 2 either underwent total sleep deprivation (*n* = 7) or were allowed free sleep (*n* = 21). The magnetic resonance imaging contrast agent gadobutrol was injected intrathecally, serving as a cerebrospinal fluid tracer. Population pharmacokinetic model parameters of gadobutrol cerebrospinal fluid-to-blood clearance were utilized as a proxy of meningeal lymphatic clearance capacity and intrathecal contrast-enhanced magnetic resonance imaging as a proxy of glymphatic function. After one night of acute sleep deprivation, the plasma concentrations of amyloid-β 40 and 42 were reduced, but not the ratio, and concentrations of the other biomarkers were unchanged. The overnight change in amyloid-β 40 and 42 plasma concentrations in the sleep group correlated significantly with indices of meningeal lymphatic clearance capacity, while this was not seen for the other neurodegeneration biomarkers. However, overnight change in plasma concentrations of amyloid-β 40 and 42 did not correlate with the glymphatic marker. On the other hand, the overnight change in plasma concentration of phosphorylated tau peptide 181 correlated significantly with the marker of glymphatic function in the sleep deprivation group but not in the sleep group. The present data add to the evidence of the role of sleep and sleep deprivation on plasma neurodegeneration concentrations; however, the various neurodegeneration biomarkers respond differently with different mechanisms behind sleep-induced alterations in amyloid-β and tau plasma concentrations. Clearance capacity of meningeal lymphatics seems more important for sleep-induced changes in amyloid-β 40 and 42 plasma concentrations, while glymphatic function seems most important for change in plasma concentration of phosphorylated tau peptide 181 during sleep deprivation. Altogether, the present data highlight diverse mechanisms behind sleep-induced effects on concentrations of plasma neurodegeneration biomarkers.

## Teaser

Sleep deprivation can impact plasma concentrations of neurodegeneration biomarkers. This human study offers *in vivo* evidence suggesting that meningeal lymphatic clearance capacity has a crucial role in sleep-induced changes in amyloid-β 40/amyloid-β 42 plasma concentrations, while glymphatic function may be particularly significant in altering the plasma concentration of phosphorylated tau peptide 181 during sleep deprivation.

## Graphical Abstract

**Graphical Abstract:**
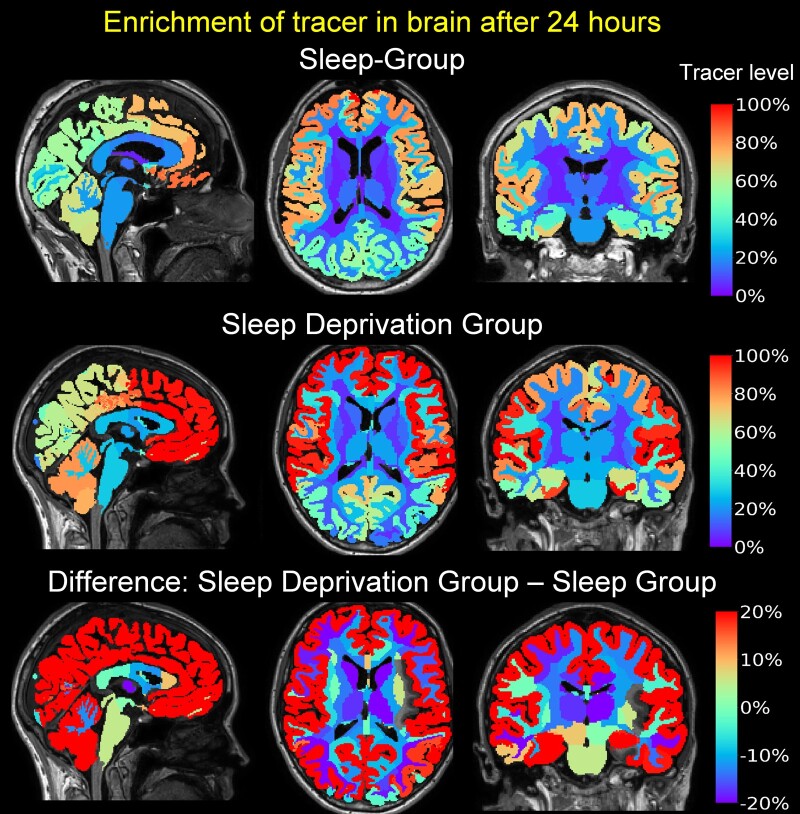

## Introduction

Accumulation of toxic waste products from brain metabolism characterizes several neurodegeneration diseases such as Alzheimer’s disease [amyloid-β (Aβ) and phosphorylated tau (P-Tau)] and Parkinson’s disease (α-synuclein),^1^ normal pressure hydrocephalus (Aβ and P-Tau)^2,3^ and dementia after traumatic brain injury (Aβ and P-Tau).^4^ The aggregation of metabolites starts years before the clinical phenotype appears and is affected by various risk factors, such as sleep disturbance.^5^ Hence, sleep impairment is a well-accepted risk factor for Alzheimer’s and Parkinson’s diseases^6,7^ and for dementia after traumatic brain injury.^8^

For early detection of dementia disease, concentrations in CSF and plasma of neurodegeneration biomarkers such as Aβ40, Aβ42, P-Tau181, glial fibrillary acid protein (GFAP) and neurofilament light (NfL) have been determined.^9-11^ These biomarker concentrations may be affected by sleep disturbance and day–night cycle,^12^ but the literature is inconsistent on how sleep impairment affects concentrations of plasma and CSF neurodegeneration biomarkers.^13-20^ Various mechanisms may be involved since toxic brain metabolites are cleared from the brain via different routes, including cellular degradation in the brain, transport across the blood–brain barrier (BBB) and egress via perivascular (glymphatic) pathways and via meningeal lymphatic pathways.^21,22^ Sleep deprivation affects BBB function,^23,24^ as well as glymphatic^25^ and meningeal lymphatic functions.^26^ To this end, the mechanistic underpinnings how acute sleep deprivation affects the plasma neurodegeneration biomarker concentrations have not been determined.

This present study was undertaken to examine how changes in plasma concentrations of neurodegeneration biomarkers induced by sleep deprivation associate with indices of meningeal lymphatic and brain glymphatic functions. We measured longitudinally plasma concentrations of Aβ40, Aβ42, P-Tau181, GFAP and NfL in a cohort of individuals who, for one night, either underwent acute sleep deprivation and, in a group, were allowed free sleep. Intrathecal contrast-enhanced MRI was done using the MRI contrast agent gadobutrol as a CSF tracer. The CSF-to-blood clearance of gadobutrol was estimated using a population pharmacokinetic model as a proxy for meningeal lymphatic clearance capacity,^27^ and brain enrichment of this extra-vascular CSF tracer was used as a proxy of glymphatic function.^28^ We previously reported increased CSF tracer levels in the brains of individuals undergoing acute sleep deprivation,^29^ as well as individuals reporting subjective chronic sleep impairment.^30^

## Materials and methods

### Permissions

These authorities approved the study: The Regional Committee for Medical and Health Research Ethics (REK) of Health Region South-East, Norway (2015/96); The Institutional Review Board of Oslo University Hospital (2015/1868); and The National Medicines Agency (15/04932-7). The study was registered in Oslo University Hospital Research Registry (ePhorte 2015/1868). Ethical standards according to the Helsinki Declaration (1975 and as revised in 1983) were followed. Following written and oral informed consent, participants were included.

### Study cohort

The participants included in this report were recruited from a prospective research study, that incorporate consecutive patients undergoing intrathecal contrast-enhanced MRI as part of their workup of CSF diseases within the Department of Neurosurgery at Oslo University Hospital, Norway. Intrathecal gadobutrol is administered off-label on clinical indication; therefore, healthy individuals were not included.

### Experimental design

The experimental design was prospective and observational. An intervention group (sleep deprivation group) underwent total sleep deprivation through 24 h from Day 1 to Day 2. A control group (sleep group), matched with the intervention group according to age and gender and randomly selected from the study population prior to analysis of MRI and FreeSurfer data, was allowed unrestricted sleep through the study period (sleep group). Hence, while the sleep group typically slept from about 10–11 pm Day 1 until about 7 am Day 2, the sleep deprivation group had no sleep from evening Day 1 to morning Day 2. The neurosurgical nursing staff observed the sleep deprivation subjects. In addition, a close relative stayed with the participant during the night to help them stay awake. They were allowed to move freely during the night but avoided caffeine to stay awake.

Intrathecal injection of the MRI contrast agent gadobutrol was done on the morning of Day 1. Venous blood samples and MRI acquisitions were obtained at multiple time points during Days 1 and 2.

### Plasma concentrations of brain metabolites

We sampled venous blood at multiple time points and stored them in a refrigerator (4°C) for a few hours, before the samples were centrifugated, aliquoted and stored in an ultrafreezer (−80°C). Plasma biomarker concentrations were measured using digital, bead-based and ultrasensitive sandwich enzyme-linked immunosorbent assays on a single molecule array HD-X-analyser utilizing the Human Neurology 4-Plex E assay for Aβ40, Aβ42, GFAP and NfL (Quanterix, Billerica, MA, USA) and an in-house single molecule array assay for P-Tau181.^31^ All measurements were done in one experimental round, utilizing one batch of reagents by board-certified laboratory technicians who were blinded to the clinical data. Intra-assay coefficients of variation were below 10%.

The goal of this study was to measure group differences in plasma concentrations on Day 2, as well as overnight change in plasma concentrations from Day 1 to Day 2. We compared the mean plasma concentrations on Days 1 and 2.

### CSF-to-blood clearance from pharmacokinetic model (proxy of meningeal lymphatic clearance)

The individual CSF-to-blood clearance capacity was estimated using a previously published population pharmacokinetic model,^27,32^ comprising a two-compartmental model with first-order elimination from the central (plasma) compartment, and distribution to peripheral tissue. The model showed an overall excellent goodness of fit. Variables of this pharmacokinetic model are used as a proxy of meningeal lymphatic clearance capacity.

### Cerebral CSF tracer enrichment (proxy of glymphatic function)

We applied the MRI contrast agent gadobutrol as a CSF tracer to examine tracer enrichment in the brain as a proxy of glymphatic function. The intrathecal dose of gadobutrol was 0.5 mmol (0.5 ml of 1.0 mmol/ml gadobutrol; Gadovist, Bayer Pharma AG, Berlin, Germany). Thereafter, standardized T_1_-weighted MRI was acquired multiple times with a 3 T Philips Ingenia MRI Scanner (Philips Medical Systems, Best, the Netherlands). For all time points, equal imaging protocol settings were used to obtain sagittal 3D T_1_-weighted volume scans. The following imaging parameters were used: repetition time = ‘shortest’ (typically 5.1 ms), echo time = ‘shortest’ (typically 2.3 ms), flip angle = 8°, field of view = 256 × 256 cm and matrix = 256 × 256 pixels (reconstructed 512 × 512). A total of 184 over-contiguous (overlapping) slices with 1 mm thickness were sampled and automatically reconstructed to 368 slices (0.5 mm thickness). Each image acquisition duration was 6 min and 29 s. Moreover, for every time point, we used an automated anatomy recognition protocol based on landmark detection in MRI data (SmartExam, Philips Medical Systems, Best, the Netherlands) to secure consistency and reproducibility of the MRI slice placement and orientation and slice orientation of image stacks.

FreeSurfer software (version 6.0) (http://surfer.nmr.mgh.harvard.edu/) was used for post-processing to segment, parcellate, register and align the longitudinal data and to determine the CSF tracer-induced increase in T_1_ signal.^33^ The presence of gadobutrol in CSF or brain tissue increases the T_1_ relaxation of water that results in higher T_1_ signal intensity at the image greyscale. The T_1_ signal intensity provides a semi-quantitative estimate of the tracer concentration. Furthermore, a hybrid watershed/surface deformation procedure enables the removal of non-brain tissue,^34^ and segmentation of the cerebral cortex and subcortical white matter can be performed.^35,36^ For each patient, the MR images were used to create a median template registered to the baseline,^37^ and the MR images were registered to the corresponding template using a rigid transformation.^37^ In order to adjust for changes in the greyscale between MRI scans, the T_1_ signal unit for each time point was divided by the T_1_ signal unit of a reference region of interest (placed within the posterior part of the orbit) for the respective time point.^29^ This ratio is denoted the ‘normalized T_1_ signal units’ and corrects for baseline image greyscale changes due to automatic image scaling. Glymphatic tracer enrichment in the sleep deprivation group has been reported previously,^29^ while the sleep group was not reported before.

### Subjective sleep quality

To obtain information about the participants general sleep quality, they were asked to report their subjective sleep quality over the last months, not referring to sleep quality over the last few days when the study was performed. We used the Pittsburgh Sleep Quality Index questionnaire,^38^ utilizing a Norwegian translation.^39^ The global score has a range from 0 to 21, with higher scores indicative of poor sleep quality.

### Statistical analyses

We performed statistical analysis with SPSS version 26 (IBM Corporation, Armonk, NY, USA) and Stata/SE 17.0 (StataCrop LLC, College Station, TX, USA). Continuous data are presented as mean (standard deviation) or mean (95% confidence intervals), as appropriate. Repeated measurements were examined with linear mixed models by maximum likelihood estimation using a subject-specific random intercept and distinct residual error parameters at different points of follow-up if appropriate. A non-linear model was used to analyse daytime variation in biomarker concentrations. For repeated measurements of the same subject, we used a fractional polynomial linear regression with a maximum of one degree of the fractional polynomial and robust standard error. Plots were presented with the linear prediction (estimated mean from the regression model) and 95% confidence interval. The Pearson correlation test was used to test correlations between different variables. Statistical significance was accepted at the 0.05 level (two-tailed).

## Results

### Study participants

The study included seven participants who underwent total sleep deprivation from Day 1 to Day 2 (sleep deprivation group) and 21 age- and gender-matched control participants (sleep group; Table 1). The groups were comparable for variables such as body mass index and general subjective sleep quality, assessed by the Pittsburgh Sleep Quality Index.

**Table 1:** Information about the two study groups

	Sleep group	Sleep deprivation group	Statistics
***N***	21	7	
**Sex (F/M)**	16/5	6/1	ns
**Age (years)**	41.2 ± 13.8	44.7 ± 15.7	ns
**BMI (kg/m^2^)**	30.3 ± 5.6	26.2 ± 3.7	ns
**Total PSQI score**	9.7 ± 4.2	8.0 ± 4.9	ns
**Tentative diagnoses**			
Idiopathic intracranial hypertension (*n*; %)	8 38%)	2 (29%)	
Spontaneous intracranial hypotension (*n*; %)	4 (19%)	1 (14%)	
Arachnoid cysts (*n*; %)	3 (14%)	1 (14%)	
Communicating hydrocephalus	1 (5%)	0	
Reference (*n*; %)^a^	5 (24%)	3 (43%)	

Data presented as mean ± SD. Continues data were examined by independent sample *t*-test and categorical data by Pearson chi-square test. BMI, body mass index; F, female; M, male; NS, no significant statistical differences between intervention groups; PSQI, Pittsburgh Sleep Quality Index. ^a^References are patients in whom no particular cause of symptoms was identified.

### Plasma biomarker concentrations after one night of sleep deprivation

**Figure 1:**
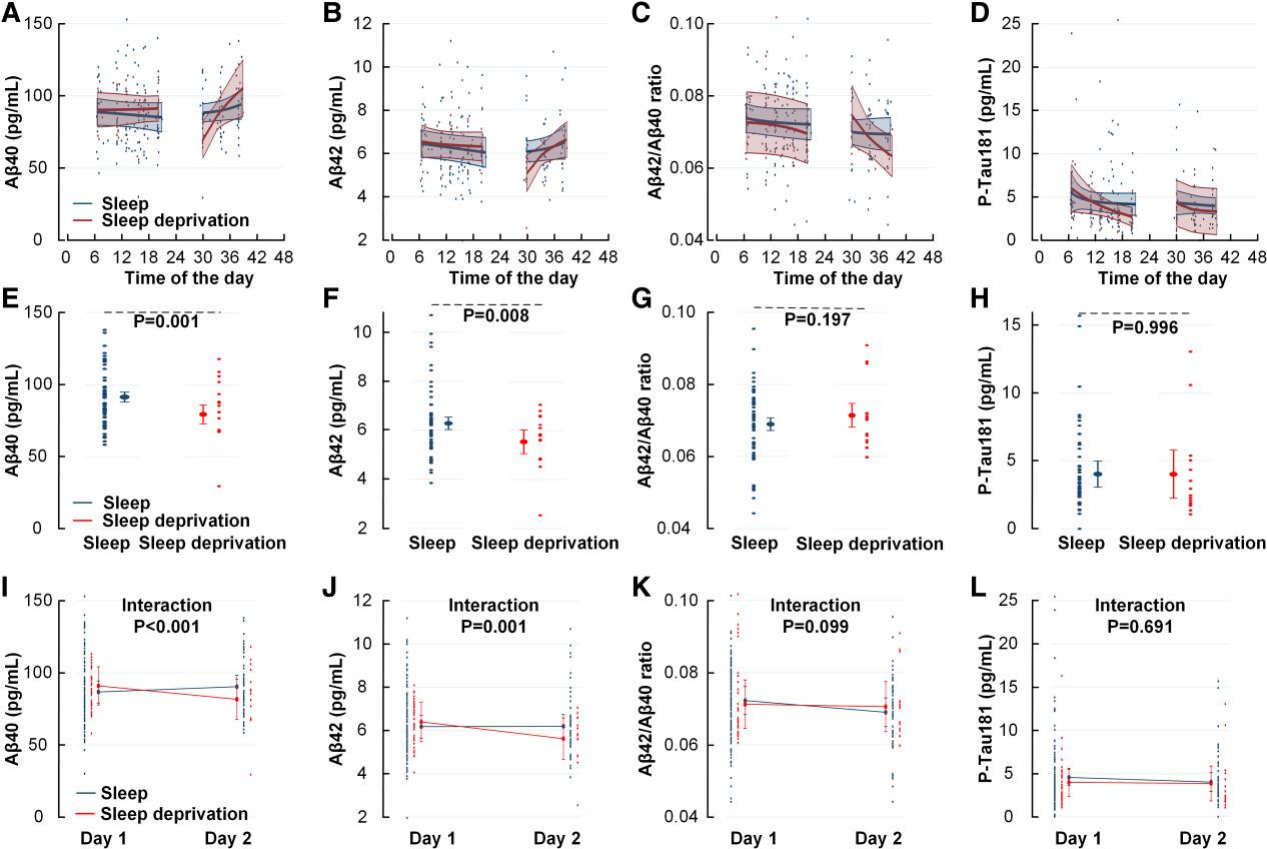
**After one night of total sleep deprivation, plasma concentrations of Aβ40 and Aβ42 are reduced, while the Aβ42/Aβ40 ratio and P-Tau181 plasma concentrations are unchanged.** (**A–D**) Differences between the sleep and sleep deprivation groups in daytime plasma concentrations of longitudinally collected plasma samples [using a non-linear model, a fractional polynomial linear regression with a maximum of one degree of the fractional polynomial and robust standard error for repeated measurements of the same subject. The *P*-value of the difference between groups depends on the specific time of the day. Therefore, plots are presented with the linear prediction (estimated mean from the regression model) and 95% confidence interval without *P*-values] for (**A**) Aβ40, (**B**) Aβ42, (**C**) Aβ42/Aβ40 ratio and (**D**) P-Tau181. (**E–H**) Comparisons of plasma concentrations [using a linear mixed model with subject-specific random intercept adjusted for mean differences between groups at Day 1. The plots report the estimated mean and 95% confidence interval from the statistical model and all single data points at Day 2. A subject may have several data points] of (**E**) Aβ40 (*P* = 0.001), (**F**) Aβ42 (*P* = 0.008), (**G**) Aβ42/Aβ40 ratio (*P* = 0.197) and (**H**) P-Tau181 (*P* = 0.996) at Day 2 between sleep and sleep deprivation groups statistically adjusted to equal concentration at Day 1. (**I–L**) Interaction between plasma concentrations Day 1 and Day 2 [using a linear mixed model with subject-specific random intercept and interaction between groups and day. The plots report the estimated mean and 95% confidence interval from the statistical model and all single data points at Days 1 and 2. A subject may have several data points at Days 1 and 2] of (**I**) Aβ40 (*P* < 0.001), (**J**) Aβ42 (*P* = 0.001), (**K**) Aβ42/Aβ40 ratio (*P* = 0.099) and (**L**) P-Tau181 (*P* = 0.691) for the sleep and sleep deprivation groups.

First, we compared the intervention groups for plasma concentrations of the neurodegeneration biomarkers the morning after sleep deprivation/sleep. The plasma concentrations of Aβ40 and Aβ42 were significantly reduced after one night of total sleep deprivation, but the Aβ42/Aβ40 ratio and P-Tau181 concentrations were unchanged (Fig. 1; Table 2). Moreover, GFAP and NfL plasma concentrations were unchanged on Day 2 after sleep deprivation (Supplementary Fig. 1).

**Table 2:** Plasma concentrations of neurodegeneration biomarkers in the sleep and sleep deprivation groups Day 2 after intervention

	Sleep group	Sleep deprivation group	Significance
Aβ40 (pg/mL)	91.6 ± 1.8	79.6 ± 3.3	*P* = 0.001
Aβ42 (pg/mL)	6.3 ± 0.1	5.5 ± 0.3	*P* = 0.008
Aβ42/Aβ40 ratio	0.069 ± 0.001	0.071 ± 0.002	ns
P-Tau181 (pg/mL)	4.02 ± 0.49	4.02 ± 0.90	ns
GFAP (pg/mL)	39.0 ± 2.3	37.9 ± 4.3	ns
NfL (pg/mL)	8.5 ± 0.2	8.5 ± 0.4	ns

Data presented as mean ± SE. ns, non-significant. Independent sample *t*-test.

### Meningeal lymphatic clearance capacity versus overnight change in plasma biomarker concentrations

**Figure 2:**
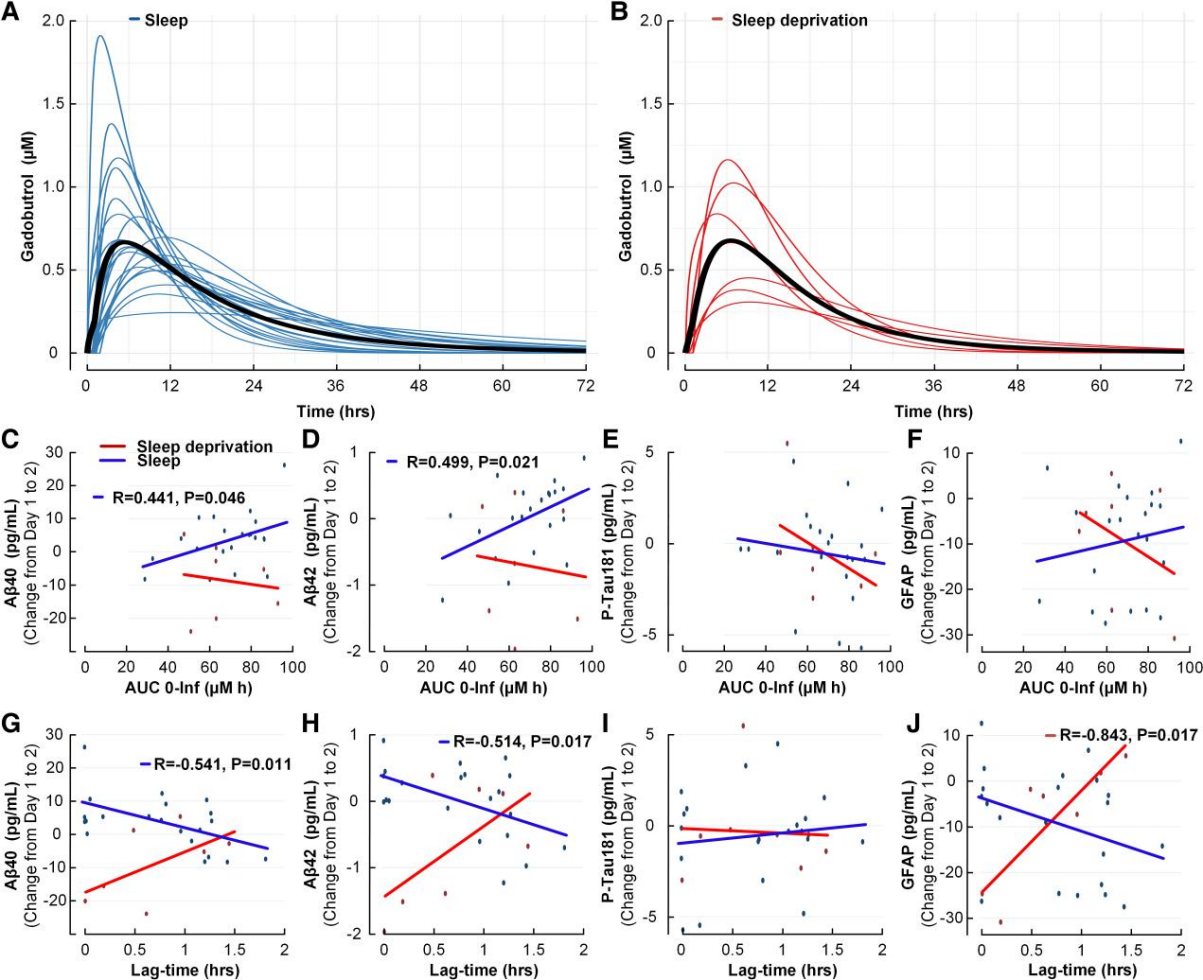
**Meningeal lymphatic clearance capacity, estimated from the CSF-to-blood clearance pharmacokinetic model, associates with overnight change in plasma concentrations of Aβ40, Aβ42 and GFAP.** (**A**, **B**) Both the sleep and sleep deprivation groups showed inter-individual variation in CSF-to-blood clearance; the individual posterior dose-normalized predicted concentrations of plasma gadobutrol over time are shown for the sleep (**A**) and sleep deprivation (**B**) groups, and the group-wise mean is shown in black. (**C–F**) In the sleep group (blue lines and dots) but not the sleep deprivation group (red lines and dots), increasing pharmacokinetic model-derived area under the curve was significantly and positively associated with a more pronounced increase in overnight plasma concentration of (**C**) Aβ40 and (**D**) Aβ42, which was not seen for overnight change in plasma concentrations of (**E**) P-Tau181 or (**F**) GFAP. (**G**, **H**) In the sleep group (blue line and dots) but not the sleep deprivation group (red line and dots), increasing pharmacokinetic model-derived lag time was correlated with less increase in (**G**) Aβ40 and (**H**) Aβ42, but not for (**I**) P-Tau181, but (**J**) overnight change in GFAP was positively correlated with longer lag time. Each plot presents the fit line and the Pearson correlation coefficient (*R*) with *P*-value.

The plasma pharmacokinetics of intrathecally administered gadobutrol determined from our population pharmacokinetic model, utilized as a proxy of meningeal lymphatic clearance capacity, was comparable between the two groups (Fig. 2A and B; Table 3). As such, sleep did not appear to have an impact on the overall clearance of tracer from CSF to blood. However, in the sleep group, as opposed to the sleep deprivation group, this proxy of meningeal lymphatic capacity associated with overnight changes in Aβ40 and Aβ42 plasma concentrations. Hence, the area under the plasma concentration time curve of gadobutrol correlated positively with overnight change in concentrations of Aβ40 and Aβ42 in the sleep group but not in the sleep deprivation group (Fig. 2C and D). This was not seen for P-Tau181 (Fig. 2E) or GFAP (Fig. 2F). Furthermore, the longer the time before initiation of clearance from CSF (i.e. longer model-estimated lag time), the less change in overnight Aβ40 and Aβ42 plasma concentrations in the sleep group, while not in the sleep deprivation group (Fig. 2G and H). This implies that in sleep, the overnight changes in plasma concentrations of Aβ40 and Aβ42 become less when the clearance process of intrathecally administered gadobutrol from CSF is delayed. This was not seen for overnight change in P-Tau181 plasma concentration (Fig. 2I). In sleep-deprived subjects, longer time before initiation of clearance from CSF (lag time) was associated with a more pronounced overnight increase in plasma GFAP concentration (Fig. 2J).

**Table 3:** CSF-to-blood clearance variables for the two treatment groups

CSF-to-blood clearance parameters	Sleep group	Sleep deprivation group
Absorption half-life (T_1/2, abs_)	3.5 ± 1.8	3.8 ± 2.1
Lag time (T_lag_)	0.82 ± 0.74	0.70 ± 0.53
Area under the curve	67.8 ± 20.2	66.5 ± 17.1
Maximum concentration (*C*_max_)	3.9 ± 2.3	3.4 ± 1.6
Time to maximum concentration (*T*_max_)	7.6 ± 3.0	7.1 ± 1.7
**Renal clearance**		
GFR (ml/min/1.73 m^2^)	96.1 ± 14.1	103.7 ± 10.4

Data presented as mean ± SD. There were no significant differences between intervention groups. Independent sample *t*-test.

### Glymphatic function versus overnight change in plasma biomarker concentrations

**Figure 3:**
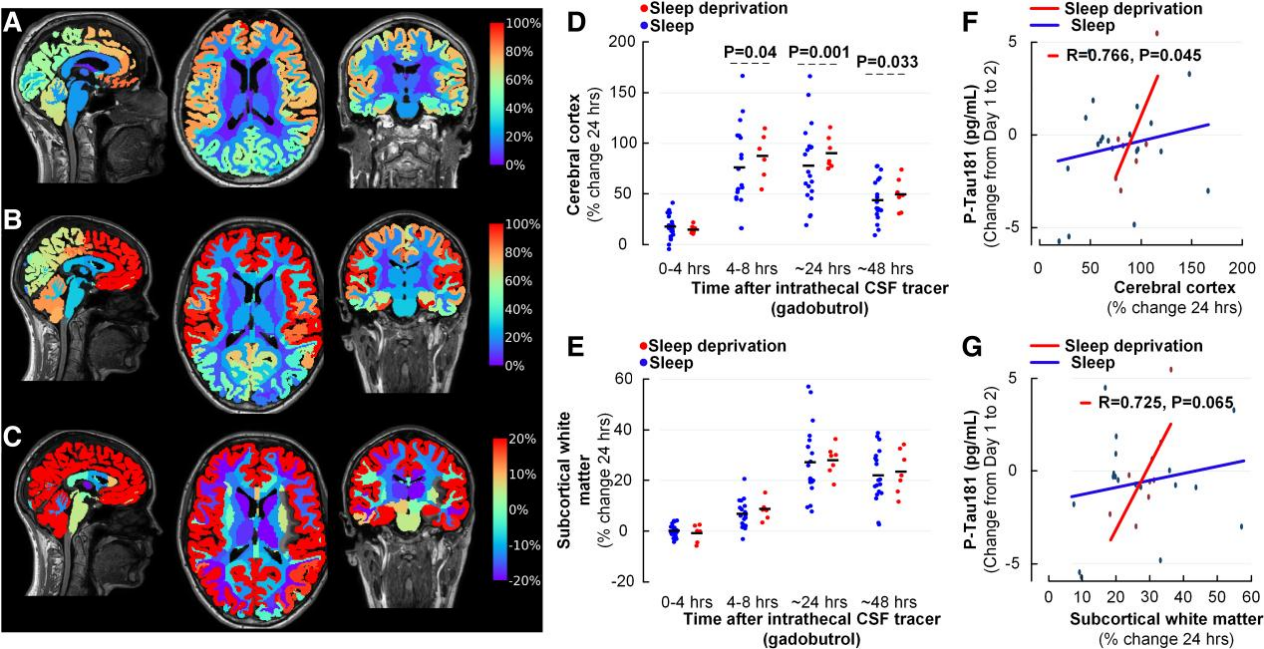
**After one night of total sleep deprivation, CSF tracer enrichment is increased in the cerebral cortex indicative of impaired glymphatic function, and the increase in CSF tracer enrichment correlates with the overnight change in plasma concentrations of P-Tau181.** (**A–C**) Color maps of CSF tracer enrichment within brain tissue at 24 h after subtraction of tracer in CSF spaces are shown for (**A**) average of sleep group, (**B**) average of sleep deprivation group and (**C**) the difference in tracer enrichment (sleep deprivation minus sleep groups). Tracer enrichment in brain tissue is expressed by percentage increase in normalized MRI T_1_ signal at 24 h as compared with baseline. Sagittal (left), axial (middle) and coronal (right) MRI scans are shown with the percentage increase in normalized T_1_ signal from baseline indicated at the color scale. Red color represents areas with the highest tracer levels. (**D**, **E**) The individual percentage changes in tracer after 0–4 h, 4–8 h, 24 h, 48 h and 4 weeks are shown for (**D**) cerebral cortex and (**E**) subcortical white matter. Sleep deprivation was accompanied with significantly higher tracer enrichment in the cerebral cortex, indicative of impaired clearance of tracer (glymphatic failure). Data shown as mean and individual levels; significance levels from linear mixed models. (**F–G**) In the sleep deprivation group (red lines and dots) but not the sleep group (blue lines and dots), there were significant positive correlations between overnight increase in plasma P-Tau181 concentrations and change in tracer enrichment in (**F**) cerebral cortex and (**G**) subcortical white matter, indicating that impaired clearance of tracer (i.e. impaired glymphatic function) is associated with a more pronounced overnight increase in plasma P-Tau181 concentration. Each plot presents the fit line and the Pearson correlation coefficient (*R*) with *P*-value.

One night of total sleep deprivation resulted in significantly reduced clearance of tracer from the cerebral cortex, indicative of impaired glymphatic (perivascular) tracer clearance (Fig. 3A–D), while clearance of tracer from subcortical white matter did not differ (Fig. 3E). Clearance of tracer from CSF was, however, not altered by sleep deprivation (Supplementary Fig. 2).

Reduced tracer clearance from the brain in sleep-deprived subjects, indicative of impaired glymphatic function, did not associate with altered Aβ40 and Aβ42 plasma concentrations (Supplementary Fig. 3). On the other hand, in the sleep deprivation group, there was a significant positive correlation between the overnight increase in P-Tau181 plasma concentration and degree of reduced tracer clearance from the cerebral cortex (Fig. 3F), while this was non-significant in the subcortical white matter (Fig. 3G). Therefore, measures of glymphatic function correlated with overnight change in plasma concentration of P-Tau181 but not with those of Aβ40 or Aβ42. With regard to overnight changes in GFAP or NfL, there were no associations with the tracer enrichment in brain after 24 h (Supplementary Fig. 4).

**Figure 4:**
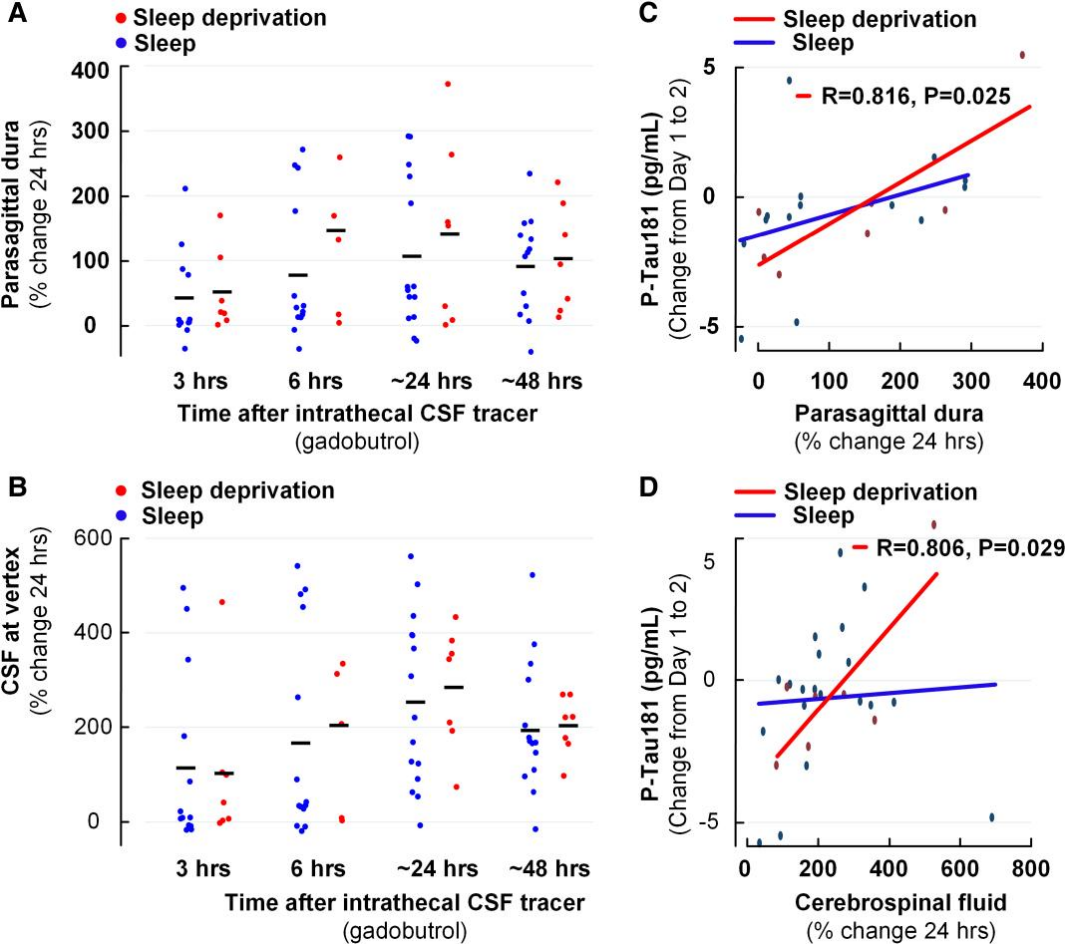
**After one night of total sleep deprivation, CSF tracer enrichment is unchanged in parasagittal dura and nearby CSF, but the change in CSF tracer enrichment correlates with the overnight change in plasma concentration of P-Tau181.** (**A**, **B**) The individual percentage changes in tracer after 3, 6, 24 and 48 h are shown for (**A**) parasagittal dura and (**B**) nearby CSF. Sleep deprivation was not accompanied with significantly altered tracer enrichment in any of the locations. Data shown as mean and individual levels; significance levels determined from linear mixed models. (**C**, **D**) In the sleep deprivation group (red lines and dots) but not in the sleep group (blue lines and dots), there were significant positive correlations between overnight increase in plasma P-Tau181 concentrations and change in tracer enrichment in (**C**) parasagittal dura and (**D**) nearby CSF, indicating that impaired clearance of tracer from these locations is associated with a more pronounced overnight increase in plasma P-Tau181 concentration. Each plot in **C** and **D** presents the fit line and the Pearson correlation coefficient (*R*) with *P*-value.

We also examined how changes in plasma concentrations of neurodegeneration biomarkers associated with tracer enrichment in CSF and the parasagittal dura. Twenty-four hours after its injection, enrichment of CSF tracer did not differ between the sleep deprivation and sleep groups in parasagittal dura (Fig. 4A) or CSF (Fig. 4B), but the increase in overnight P-Tau181 plasma concentration correlated significantly with tracer enrichment in parasagittal dura (Fig. 4C) and nearby CSF (Fig. 4D). Comparable correlations between tracer enrichment and overnight change in Aβ40 and Aβ42 plasma concentration were not found (Supplementary Fig. 5). There was neither any significant association between tracer enrichment in parasagittal dura or CSF and overnight change in plasma concentrations of GFAP or NfL (Supplementary Fig. 6).

## Discussion

The present results address possible mechanisms by which sleep and sleep deprivation affect plasma concentrations of neurodegeneration biomarkers. Reduced plasma concentrations of Aβ40 and Aβ42 after one night of acute sleep deprivation could be caused by impaired meningeal lymphatic clearance capacity of Aβ40 and Aβ42. The overnight change in plasma concentrations of Aβ40 and Aβ42 correlated with indices of meningeal lymphatic clearance capacity in sleeping, but not sleep-deprived, subjects. Plasma concentrations of P-Tau181, GFAP and NfL remained unchanged after sleep deprivation. However, in the sleep deprivation group, there was an overnight increase in P-Tau181 plasma concentrations that correlated positively with the increased tracer enrichment after 24 h in the brain, a measure of impaired glymphatic function. This indicates a closer association between P-Tau181 and glymphatic function. Clearance of the neurodegeneration biomarkers GFAP or NfL seemed less consistently affected by sleep deprivation.

As compared with the present results, acute sleep deprivation was previously reported to reduce plasma concentrations of Aβ40 and Aβ42,^14^ but others found no significant reduction.^13^ Several researchers have reported increased plasma concentrations of P-Tau181 after sleep deprivation.^13,15,18^ Reduced P-Tau181 plasma concentrations have also been reported after sleep deprivation.^14^ Here, the P-Tau181 plasma concentrations did not differ between intervention groups on the morning Day 2. Similar to the present observations, acute sleep deprivation was not found to affect plasma concentrations of GFAP or NfL.^13^ These various effects of sleep deprivation on plasma concentrations of neurodegeneration biomarkers may indicate diverse underlying mechanisms. In this regard, it should be noted that both sleep and circadian rhythm affect the various egress routes for neurodegeneration biomarkers, such as transport across the BBB,^23,24^ and via glymphatic^40-42^ and meningeal lymphatic^26,41^ pathways.

This study specifically addressed meningeal lymphatic and glymphatic clearance, utilizing a CSF tracer (gadobutrol), which is a hydrophilic molecule with a molecular weight of 604 Da (hydraulic diameter about 2 nm) that distributes freely within the extra-vascular compartment of the brain, largely not passing the BBB.^28^ We have previously suggested that the population pharmacokinetic model-estimated CSF-to-blood clearance variables provide an overall measure of meningeal lymphatic clearance capacity.^27,32^ This assumption is supported by previous observations of passage of the tracer from CSF to the parasagittal dura,^43^ skull bone marrow^44^ and extra-cranial lymph nodes.^45^ It was recently verified that human dura mater harbours lymphatic vessels.^46^ Moreover, the arachnoid granulations traditionally considered to be passive CSF passage routes to the dural venous sinuses may be additional pathways to meningeal lymphatic structures.^47^ Therefore, the pharmacokinetic-estimated CSF-to-blood clearance variables may depict the overall meningeal lymphatic clearance capacity. Exceptions are conditions with disrupted BBB or CSF leakage where the CSF-to-blood clearance variables incorporate an overall estimate of CSF-to-blood clearance capacity. As shown here (Fig. 2), the CSF-to-blood clearance capacity varies extensively between subjects,^27^ which contributes to the variability within treatment groups.

The glymphatic pathway was conceptualized as a perivascular pathway for the convective transport of fluids and solutes along the arterial brain vessels, via interstitial tissue and with efflux along the venous brain vessels being primarily active during sleep.^25,40^ Several aspects of the glymphatic concept are still heavily debated with no generally accepted methods to assess its function in humans. Here, the term ‘glymphatic’ refers to the perivascular solute transport of the glymphatic concept. The intrathecal contrast-enhanced MRI may currently be considered gold standard for human *in vivo* glymphatic imaging based on the following: (i) the CSF tracer is transported antegrade along arteries but is confined outside vessels due to the BBB^48^; (ii) the CSF tracer transport is faster than extra-cellular diffusion^49^; (iii) the CSF tracer enriches brain tissue centripetal from outside and inward^28^; and (iv) the cerebral tracer enrichment is sleep dependent, being altered both by acute sleep deprivation^29^ and chronic impaired sleep quality.^30^ In support of this, we recently reported that plasma concentrations of neurodegeneration biomarkers correlate with enrichment of the CSF tracer in the brain and CSF.^12^

An increasing body of evidence suggests that the meningeal lymphatic vessels are crucial for the egress of Aβ from CSF to blood^50^ via extra-cranial lymph nodes.^51,52^ Impaired meningeal lymphatic function also aggravates anti-Aβ immunotherapy.^53^ The present observations provide another perspective to the role of meningeal lymphatic clearance function for Aβ clearance. In sleeping subjects, the overnight change in Aβ40 and Aβ42 plasma concentration correlated significantly with pharmacokinetic-estimated CSF-to-blood clearance variables. However, this relationship was disturbed in sleep-deprived subjects who presented with reduced Aβ40 and Aβ42 plasma concentrations on the morning Day 2. These findings add to the evidence that meningeal lymphatic clearance function is a significant contributor to Aβ clearance, even though there are various Aβ clearance routes from the brain via BBB and CSF (glymphatic and meningeal lymphatic pathways).^21,51,54^ For example, Aβ passes from the brain across the BBB via P-glycoprotein and lipoprotein receptor-related protein-1 transporters^55-57^; P-glycoprotein activity is diurnal though seems not to be sleep dependent.^58^

While overnight changes in plasma concentrations of Aβ40 and Aβ42 were not associated with tracer enrichment after 24 h in either sleep deprivation or sleep groups, clearance of P-Tau181 correlated with the glymphatic marker. In the sleep deprivation group, there was a significant positive correlation between the overnight increase in P-Tau181 plasma concentrations and the increased tracer enrichment in the cerebral cortex at 24 h (i.e. proxy of impaired glymphatic function). This finding aligns with our recent observations of a close association between plasma P-Tau concentrations and glymphatic function assessed by intrathecal contrast-enhanced MRI.^12^ Others previously provided experimental evidence for a pivotal role of aquaporin-4-dependent glymphatic function for tau clearance from the brain.^59^ In addition, we here showed a positive correlation between P-Tau181 increase and the increased tracer enrichment in parasagittal dura and nearby CSF. It might seem like a paradox that impaired clearance of P-Tau181 from the brain and CSF was associated with an increase in overnight plasma concentration. One possible explanation is that sleep deprivation increases molecular passage via BBB and thereby increases plasma P-Tau181 concentrations. Tau has a BBB transporter,^60^ and BBB integrity is under sleep and circadian control.^24^

Some limitations of the study should be noted. This study included consecutive patients who were willing to stay awake for one night, without further selection criteria. To which degree the group who accepted to stay awake is a biased group is unknown. However, the subjective sleep quality measured according to the Pittsburgh Sleep Quality Index was not different between the groups (Table 1).

For *in vivo* assessment of glymphatic function, we utilize an MRI contrast agent as a CSF tracer, based on a hypothesis that CSF tracer enrichment is indicative of the extra-vascular transport of soluble metabolic waste products such as Aβ, tau and α-synuclein. The presently used CSF tracer is hydrophilic, confined primarily outside the blood vessels but with several times smaller molecular size than the metabolites. Molecular weights of the presently addressed substances are as follows: adobutrol (604 Da), Aβ40 (4.3 kDa), Aβ42 (4.5 kDa), tau (80 kDa), GFAP (50 kDa) and NfL (70 kDa). The molecular size *per se* may not be limiting since the distribution of a CSF tracer (AlexaFluor647-conjugated bovine serum albumin) with a molecular size of 66 kDa, similar to Aβ and tau, was comparable in pig gyrencephalic brain and human brain and with documentation of perivascular tracer distribution.^61^ Furthermore, our previous observations of significant correlations between plasma concentrations of metabolites such as tau and CSF tracer enrichment^12^ strengthen the reliability of utilizing contrast agents as tracers for glymphatic function.

It may also be considered a limitation that the population pharmacokinetic model-based estimate of CSF-to-blood clearance^27^ does not define the clearance route such as via BBB or meningeal lymphatic pathways. For substances excreted directly from CSF, the primary route most likely is via meningeal lymphatic structures. Accumulating evidence suggests a crucial role of meningeal (dural) lymphatic vessels for the efflux route of solutes from intracranial CSF spaces,^62^ where the parasagittal dura is bridging the link between subarachnoid CSF spaces and the dural lymphatic vessels in humans.^43^ Lymphatic drainage was faster in awake than anesthetized mice,^26^ suggesting that lymphatic efflux to extra-cranial lymph nodes is enhanced during the awake state.

It is presently not clear how differences in clearance kinetics of the presently reported substances relate to the proxies of glymphatic and meningeal lymphatic functions. The CSF-to-blood clearance variable ‘absorption half-life’ (T_1/2, abs_) of gadobutrol was 3.5 ± 1.8 h in the sleep group and 3.8 ± 2.1 h the in sleep deprivation group (Table 3; Fig. 2). In comparison, the half-life of Aβ depends somewhat on isoform but was approximately 3 h in plasma,^63^ as compared with about 9 h in CSF.^64^ In comparison, the half-life of tau in the brain is about 3 weeks,^65,66^ while about 10 h both in CSF and plasma.^65^ Further studies need to understand how the kinetics of the exogenous tracer reflect the kinetics of endogenous substances.

Finally, it may also be considered a limitation that the CSF tracer, gadobutrol, is administered off-label. For this reason, healthy subjects were not included; the present patients were examined for tentative CSF disorders. Subjects denoted references did not receive a diagnosis after clinical workup. While none of the current subjects had dementia and did not suffer severe conditions, they cannot be considered strictly as healthy individuals. There was no clinical indication for cognitive testing in the presently reported individuals. Even though intrathecal gadobutrol is administered off-label, we found no evidence of severe adverse events in three different safety studies,^67-69^ and MRI T_1_ mapping 4 weeks after intrathecal gadobutrol gave no evidence for retention of gadobutrol in the -brain.^70^ On this background, we are confident that intrathecal gadobutrol in a dose of 0.50 mmol or below is safe.

## Conclusion

In conclusion, the present results highlight the diverse mechanisms by which sleep deprivation changes plasma concentrations of neurodegeneration biomarkers. The results suggest impaired meningeal lymphatic clearance function behind reduced Aβ40 and Aβ42 plasma concentrations after sleep deprivation. Impaired glymphatic function caused by sleep deprivation seemed important for the overnight increase in P-Tau181 plasma concentration. Finally, the effects of acute sleep deprivation on plasma concentrations of GFAP and NfL were minor; clearance of GFAP showed some association with meningeal clearance function.

## Supplementary Material

fcad343_Supplementary_Data

## Data Availability

The data presented in this work are available upon reasonable request.
