# Supplementary material for: Mechanisms behind changes of neurodegeneration biomarkers in plasma induced by sleep deprivation
Source: Brain Commun. 2023 Dec 12;5(6):fcad343. doi: 10.1093/braincomms/fcad343 (PMC10733810; doi:10.1093/braincomms/fcad343)
Supplement: fcad343_Supplementary_Data [file fcad343_supplementary_data.pdf]

Supplementary Figure 1

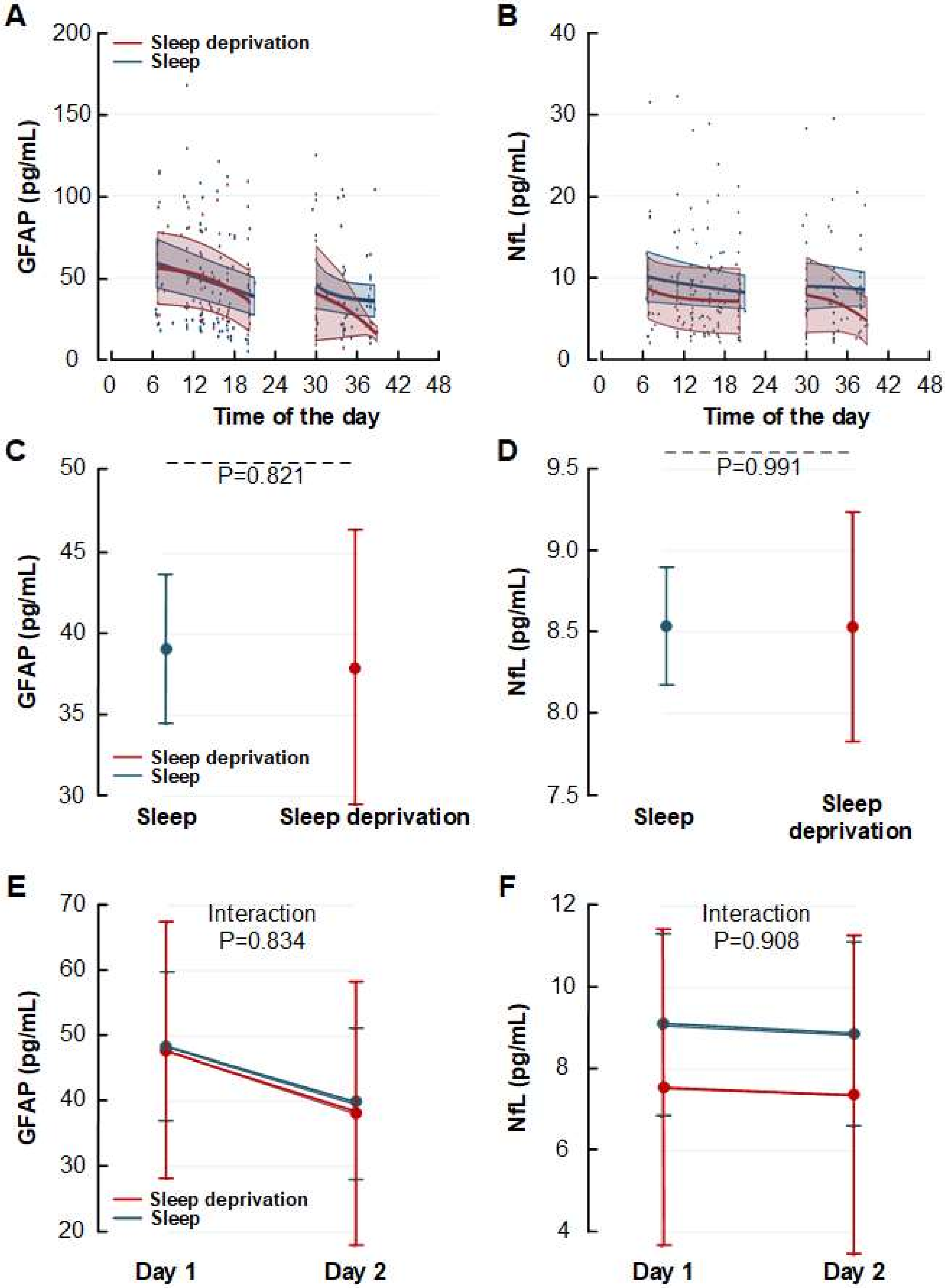

Unchanged plasma concentrations of GFAP and NfL after one night of total sleep deprivation. A-B: Differences between the Sleep and Sleep deprivation groups in daytime plasma concentrations of longitudinally collected plasma samples for (A) GFAP and (B) NfL (mean and 95%CI). **C-D:** Comparisons between Sleep and Sleep deprivation groups of plasma concentrations of (C) GFAP and (D) NfL (mean and 95%CI) morning Day 2. **E-F:** Interaction between plasma concentrations Day 1 and Day 2 of (E) GFAP and (F) NfL (mean and 95%CI) for the Sleep and Sleep deprivation groups.

Supplementary Figure 2

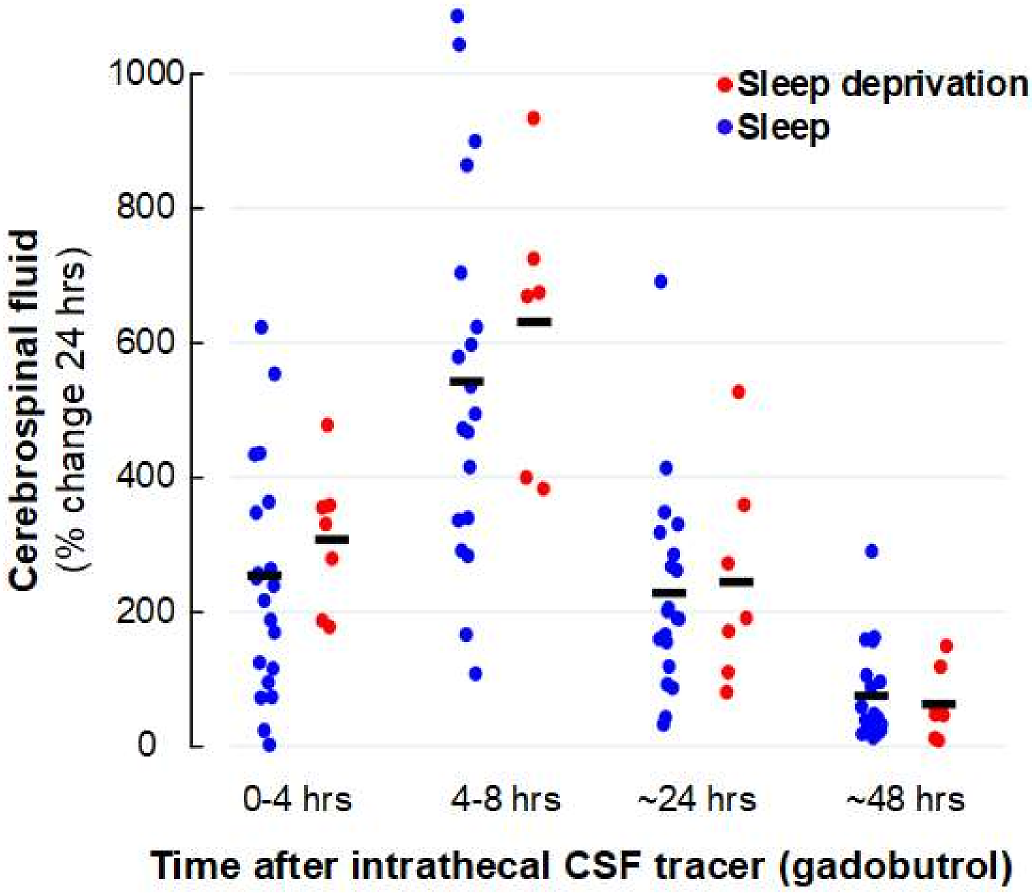

Unchanged CSF tracer enrichment in CSF after one-night of total sleep deprivation. The individual percentage changes in tracer after 0-4 hours, 4-8 hours, 24 hours, and 48 hours are shown for CSF. Sleep deprivation was not accompanied with significantly altered tracer enrichment in CSF. Data shown as mean and individual levels; significance levels from linear mixed models.

Supplementary Figure 3

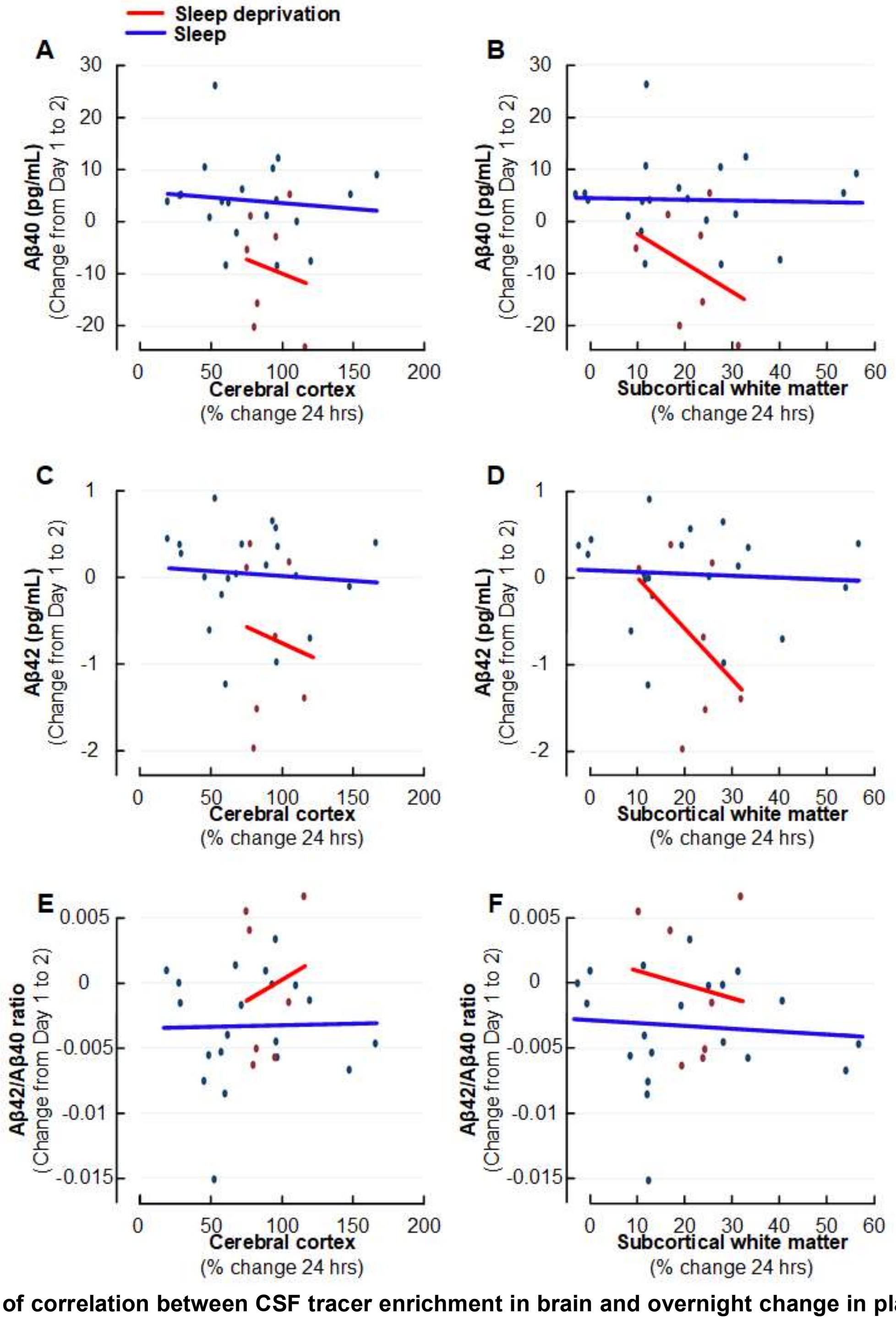

Lack of correlation between CSF tracer enrichment in brain and overnight change in plasma concentrations of Aβ40 and Aβ42. Neither the sleep deprivation group (red lines and dots), nor the sleep group (blue lines and dots), showed any significant correlations between overnight change in Aβ40 (A-B), Aβ42 (C-D) or Aβ42/ Aβ40 ratio (E-F) and CSF tracer enrichment in cerebral cortex (A, C, E), or subcortical white matter (B, D, F). Each plot presents the fit line and the Pearson correlation coefficient (R) with P-value.

Supplementary Figure 4

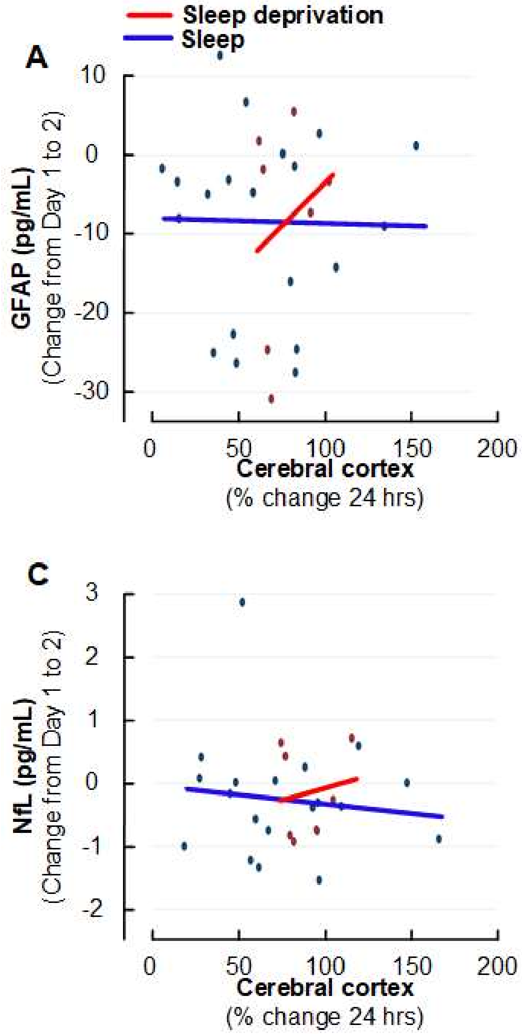

Lack of associations between CSF tracer enrichment in brain and overnight change in plasma concentrations of GFAP and NfL. Neither the sleep deprivation group (red lines and dots), nor the sleep group (blue lines and dots), showed any significant correlations between overnight change in GFAP (A-B) or NfL (C-D) and CSF tracer enrichment in cerebral cortex (A, C) or subcortical white matter (B, D). Each plot presents the fit line and the Pearson correlation coefficient (R) with P-value.

Supplementary Figure 5

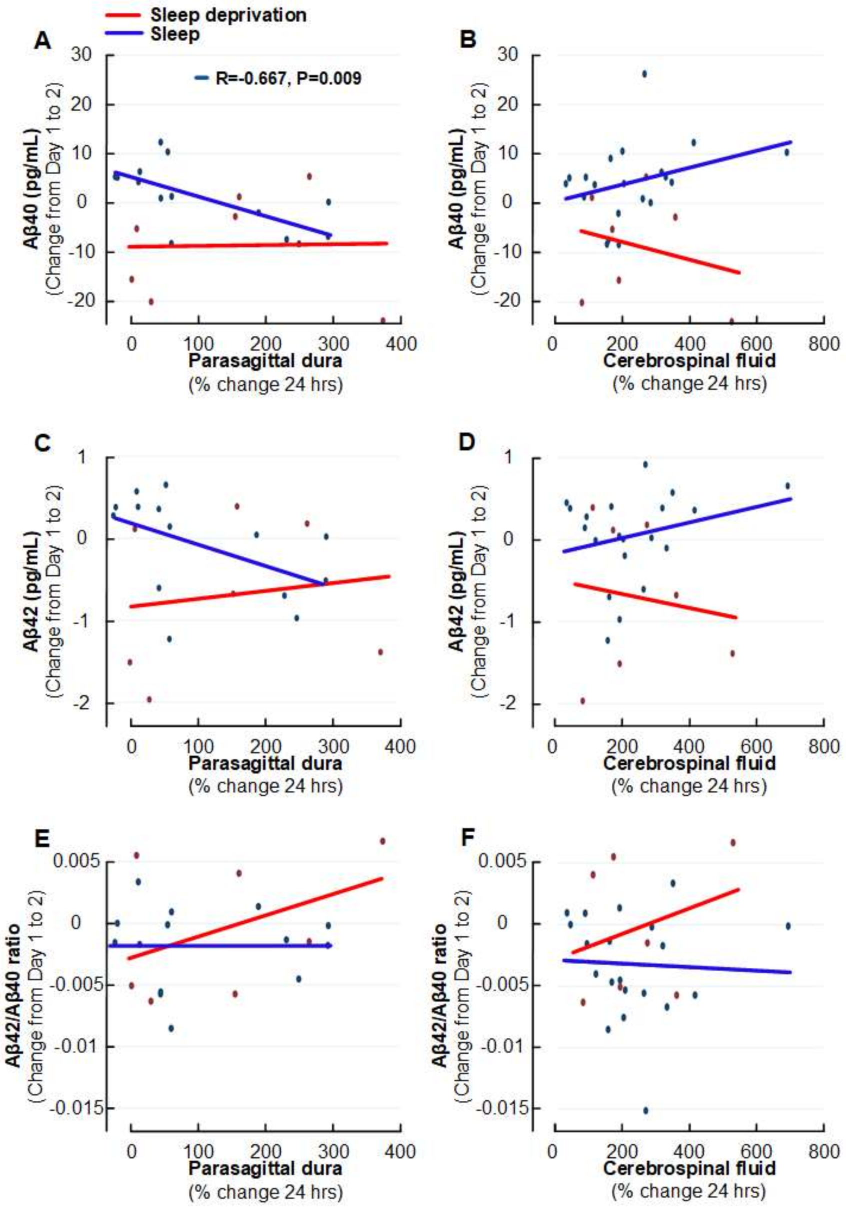

Associations between CSF tracer enrichment in parasagittal dura and nearby CSF and overnight change in plasma concentrations of Aβ40 and Aβ42. The profiles of correlations between overnight change in plasma concentrations of Aβ40 (A-B), Aβ42 (C-D) and Aβ42/ Aβ40 ratio (E-F) and CSF tracer enrichment in parasagittal dura (A, C, E) and nearby CSF (B, D, F) in the Sleep (blue lines and dots) and Sleep deprivation (red lines and dots) groups. Each plot presents the fit line and the Pearson correlation coefficient (R) with P-value. For neither of overnight change in Aβ40, Aβ42, or Aβ42/ Aβ40 ratio, there were any significant correlations between overnight change in plasma concentrations and tracer enrichment in any of the locations. The only exception was significantly less over-night change in plasma Aβ40 concentration with increasing tracer enrichment in parasagittal dura in the Sleep group, indicative of impaired clearance from parasagittal dura (A).

Supplementary Figure 6

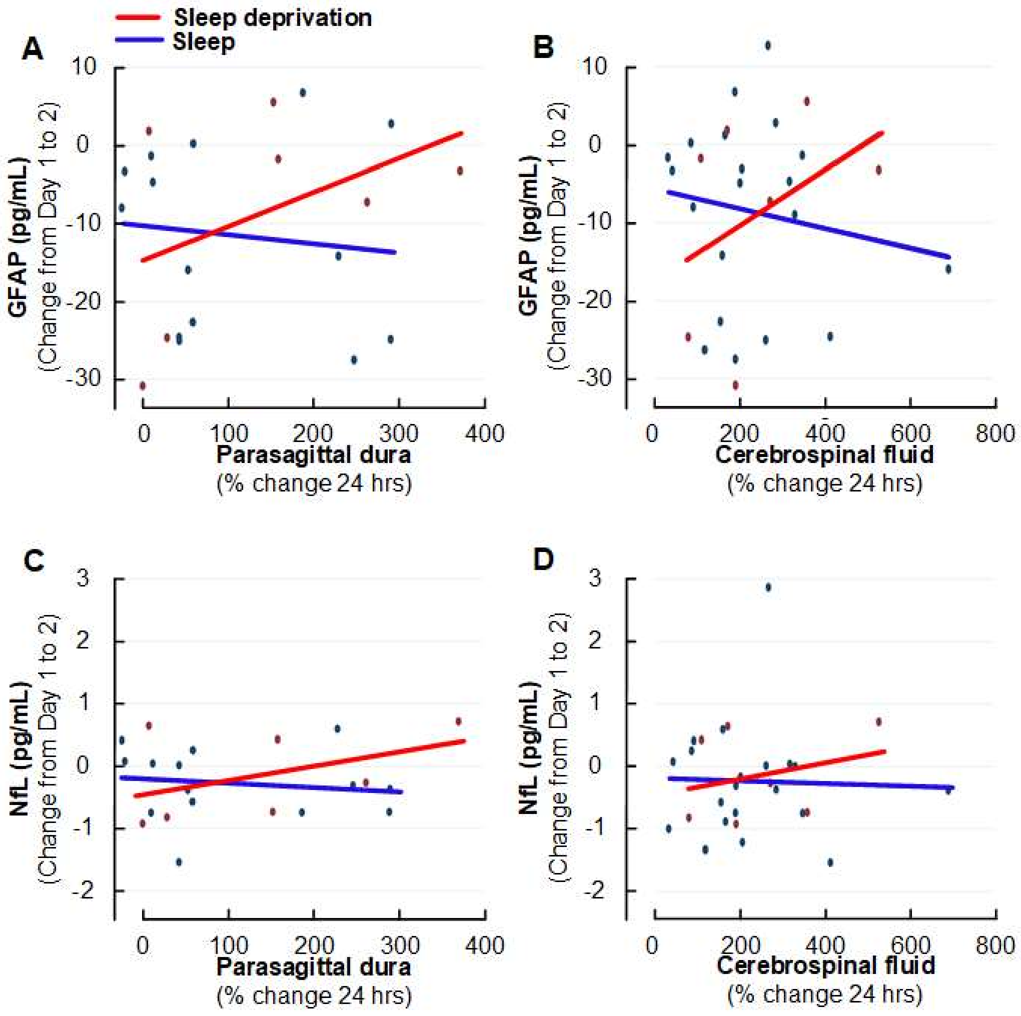

Lack of associations between CSF tracer enrichment in parasagittal dura and nearby CSF and overnight change in plasma concentrations of GFAP and NfL. The profiles of correlations between overnight change in GFAP (A-B) and NfL (C-D) and CSF tracer enrichment in parasagittal dura (A, C) and nearby CSF (B, D) in the Sleep (blue lines and dots) and Sleep deprivation (red lines and dots) groups. Each plot presents the fit line and the Pearson correlation coefficient (R) with P-value. For none of the neurodegeneration biomarkers, there were any significant correlations between overnight change in plasma concentrations and tracer enrichment.
